# Childhood trauma in schizophrenia spectrum disorders and intensity of psychotic symptoms

**DOI:** 10.1192/j.eurpsy.2021.445

**Published:** 2021-08-13

**Authors:** M.-J. Alvarez, Q. Foguet-Boreu, J. García-Eslava, P. Roura-Poch, E. Tasa-Vinyals, A. González-Vázquez, H. Masramon

**Affiliations:** 1 Mental Health Department, Consorci Hositalari Vic, Vic, Spain; 2 Epidemiology Department, Consorci Hospitalari Vic, Vic, Spain; 3 Mental Health Department, Complejo Hospitalario A Coruña, a Coruña, Spain; 4 Mental Health Department, Clínica Sant Josep, Vic, Spain

**Keywords:** Schizophrenia spectrum disorders, childhood trauma, positive psychotic symptoms, Negative psychotic symptoms

## Abstract

**Introduction:**

The relationship between history of childhood trauma (CT) and current schizophrenic symptoms is complex and controversial. Most of the studies report more positive psychotic symptoms (PPS) in psychotic patients who had suffered CT. Findings for negative psychotic symptoms (NPS) are mixed: most authors do not find differences or even find less.

**Objectives:**

The purpose of this study is to evaluate and describe the types of CT suffered by patients diagnosed with schizophrenia spectrum disorders (SSD), and to analyse the relationship between history of CT and the present-time intensity of PPS and NPS.

**Methods:**

We conducted a cross-sectional study of 45 adult patients with a SSD. Instruments: Childhood Trauma Questionnaire, short form (CTQ-SF) for measuring CT and Positive and Negative Syndrome Scale (PANSS) to assess the PPS and NPS of psychosis.

**Results:**

77.8% of the patients reported having suffered any kind of CT. By types of trauma: 48.9% reported emotional abuse, 28.9% physical abuse, 40.0% sexual abuse, 55.6% emotional neglect and 46.7% physical neglect. A lineal correlation between CTQ-SF and PANSS+/- scores was performed. Neither total PANSS+ nor any particular PANSS+ items correlate with CTQ scores. A significant inverse lineal association of moderate intensity exists between total PANSS− score and CT intensity (ρ = −0.300, p = 0.045)

**Conclusions:**

In line with previous research, our study has found inverse correlation between NPS and CT. In contrast, no association was found between PPS and CT. Our sample was mostly composed by chronic patients, which might explain the differences with the previous literature.

**Disclosure:**

No significant relationships.

